# Clinical and microbiological profile of patients with fungal keratitis demonstrating unusual yeast-like structures in potassium hydroxide with calcofluor white preparation of corneal scraping

**DOI:** 10.1186/s12348-025-00479-5

**Published:** 2025-05-26

**Authors:** Sikha Misra, Savitri Sharma, Manas Ranjan Barik, Nisha Rani, Sujata Das, Srikant Kumar Sahu, Smruti Rekha Priyadarshini, Himansu Sekhar Behera

**Affiliations:** 1 Cornea & Anterior Segment Services, L V Prasad Eye Institute, Bhubaneswar, 751024 Odisha India; 2 Ocular Microbiology Services, L V Prasad Eye Institute, Bhubaneswar, 751024 Odisha India

**Keywords:** Keratitis, Yeast, *Candida*, Budding yeast, PCR, DNA sequencing

## Abstract

**Background:**

To report a case series of microbial keratitis showing atypical yeast-like structures in direct microscopy which were culture-negative but subsequently identified as yeast cells by PCR and DNA sequencing.

**Main text:**

This is a retrospective, non-comparative case series of eight patients with infectious keratitis, where smear examination (potassium hydroxide + calcofluor white) showed spore like structures resembling yeast. There was no growth in any solid culture media. Routine PCR assay was performed using pan fungal primers followed by Sanger sequencing and nucleotide sequences were analysed using NCBI-BLAST software. Medical treatment in all patients were initiated based on clinical suspicion and presumptive microbiology report. Therapeutic penetrating keratoplasty was performed for patients not responding to medical antifungal therapy. Demographic, clinical data were collected for each patient from electronic medical records of the patients and outcome analysed. Amplification of fungal DNA was seen in the PCR assay of all samples. Nucleotide sequences of the amplicons obtained after Sanger sequencing and NCBI-BLAST analysis were found identical to *Candida albicans* (*n* = 7) and *Citeromyces matriensis* (*n* = 1). Patients were treated with antifungal drugs such as topical natamycin 5% or amphotericin B 0.15%. Ulcer resolved with scarring in 5 patients (62.5%), one patient had failed graft after therapeutic penetrating keratoplasty (12.5%), one (12.5%) eye became phthisical and one patient (12.5%) was lost to follow up.

**Conclusion:**

Atypical structures resembling yeast-like cells detected in direct smear examination in the absence of growth in culture media should raise the suspicion of a fungal etiology and warrant further investigations to establish the diagnosis.

## Introduction

Yeast-like fungi, predominantly *Candida* spp. are known to be responsible for opportunistic infections especially in immunocompromised hosts [1]. Genus *Candida* includes species such as *C. albicans*,* C. parapsilosis*,* C. glabrata*,* C. tropicalis*, and *C. krusei*. They are present as commensal in various mucosal surfaces such as skin, respiratory, digestive and urinary tract of human body [2, 3] However, these fungal species are not considered as commensal on the ocular surface. A study reported *Malassezia* (74.65%), *Rhodotorula* (1.93%), *Davidiella* (1.89%), *Aspergillus* (1.25%) and *Alternaria* (0.61%), as the ‘core fungal taxa’ on normal ocular surface with high-throughput sequencing approach [4]. Now, use of shotgun metagenomic sequencing has increased the horizon of infective keratitis diagnosis especially in culture negative microbial keratitis. It is also helpful in identifying the presence of more than one pathogen in clinical samples. It also increases the diagnostic yield as it amplifies the targeted pathogen DNA from very low sample volume [5].

Clinically *Candida* keratitis resembles bacterial or microsporidial due to discrete stromal infiltrates with overlying epithelial defect and slow progression [6]. Occasionally can progress rapidly resulting in ulceration, melting and perforation emphasising the role of appropriate microbiological evaluation [7].

Classified under yeast-like fungi, *Candida* species are known as “shape-shifter” as they change their morphology for better penetration into epithelial host cells. They can be found as unicellular budding yeast cells or filamentous form as true hyphae or pseudophyphae [8]. Pseudohyphae can be differentiated from true hyphae by absence of internal cross wall septa. This anisotropic growth from yeast to hyphal form facilitates the cellular invasion and protection from the immune system [9]. During yeast to hyphal transition the Golgi complex is shifted towards one growing hyphal tip and helps the bud in growing vis-a-vis mitosis [10].

At our tertiary eye care institute, all clinical cases of microbial keratitis undergo microbiological investigation which includes direct microscopic examination of corneal scrapings and culture sensitivity detection. In the present set of cases reported here, we observed atypical round to oval shaped structures in fluorescence microscopy of corneal scrapings under potassium hydroxide and calcofluor white (KOH + CFW) mount. These structures had an unclear resemblance to yeast cell budding, which on further investigation using molecular methods confirmed these to be either *Candida* or *Citeromyces* species. The aim of reporting this study is to increase the awareness among professionals engaged in management of microbial keratitis regarding these atypical structures.

## Materials and methods

### Study design

This is a retrospective, noncomparative case series of eight patients with suspected microbial keratitis seen at the L. V. Prasad Eye Institute, Bhubaneswar, India, between 1st January 2023 and 31st December 2023, where the initial smear report was inconclusive. The medical records of the patients were identified using the 2005 ICD-9-CM code, and retrieved from the institute’s electronic medical records (EMR) system. The institutional ethics committee approved this study (LEC-BHR-R-12-23-1159). The study was conducted in strict adherence to the tenets of the Declaration of Helsinki.

### Data collection

The demographic and clinical data of the patients were extracted from the EMR and entered manually into a predesigned spreadsheet. Demographic data included history of any systemic illness, clinical data at presentation, etiological factors (ocular trauma, contact lens use, steroid use), baseline best-corrected visual acuity, site of corneal ulcer, size of epithelial defect, stromal infiltrates and hypopyon, use of systemic medications, associated ocular comorbidities were collected. The treatment given, complications, outcomes, and duration of follow-up were also collected in detail. Visual acuity was recorded using the Snellen distance visual acuity chart. The size of the corneal ulcer was measured using a slit lamp, with a 1 mm thick slit beam in its greatest dimension and perpendicular to the greatest dimension. Ulcer was divided into grade1 to 3 depending upon its size. Less than 2 mm as grade 1, 2–5 mm as grade 2 and more than 5 mm as grade 3.

### Smear examination and culture

Under topical anaesthesia, corneal scrapings were collected in the clinic and subjected to microbiological processing as per the institute’s protocol for all patients [11, 12]. Sample was smeared on one slide for Gram staining and placed on another slide for potassium hydroxide + calcofluor white (KOH + CFW) mount; and simultaneously inoculated on different culture media, such as 5% sheep blood chocolate agar (CA), 5% sheep blood agar (BA), Sabouraud dextrose agar (SDA), non-nutrient agar with *Escherichia coli* (NNA), brain heart infusion broth (BHI), Robertson’s cooked meat medium (RCM), and thioglycollate broth (THB). SDA and PDA plates were incubated at 25 °C to facilitate the growth of fungi. The remaining media were incubated at 37 °C to facilitate the growth of bacteria.

### DNA isolation from clinical sample

DNA was extracted from the corneal scraping of all patients using QIA-amp DNA Mini Kit (QIAGEN, GmbH, Hilden (Germany), following manufacturer’s instructions. Isolated DNA from all patient samples was screened for the presence of fungal DNA with the conventional PCR assay as described below.

Simultaneously, corneal scraping collected from 12 smear (KOH + CFW mount and Gram stain) and culture negative suspected microbial keratitis patients were processed for DNA extraction using QIA-amp DNA Mini Kit following manufacturer’s instructions.

### Conventional PCR assay

Briefly, Pan fungal (ITS gene) PCR assay was carried out on DNA from all corneal scrapings including from control subjects in a T100 Thermal Cycler (BIORAD, USA) with 25µL of final reaction volume containing 5.5µL nuclease free water, 12.5µL 2x Taq PCR Master Mix (GCC BIOTECH, India), 1µL each of forward and reverse primers and 5 µL of extracted DNA. The primers used in the PCR assay were a published set of pan-fungal primers (FP: 5’GTGAAATTGTTGAAAGGGAA3’ and RP: 5’GACTCCTTGGTCCGTGTT3’**)** [13]. The PCR condition was 5 min at 94˚C, followed by 34 cycles of 30 s at 94˚C, 30 s at 58˚C, 30 s at 72˚C, with final elongation for 7 min at 72˚C. DNA extracted from known *Candida albicans* (ATCC 10231) culture was used as a positive control and reaction mixture with 5 µl of distilled water was used as a negative control in all PCR reactions. Amplified PCR products (~ 259 bp) were electrophoresed and visualized under a gel documentation system (BIORAD, USA).

### DNA sequencing

DNA sequencing was performed with Sanger’s dideoxy nucleotide method on a ABI3130 genetic analyzer (Eurofins) and sequence data was analyzed using Finch TV software. Nucleotide sequences of both DNA strands were analysed using the National Center for Biotechnology Information (NCBI) Basic local alignment search tool (BLAST) software available at http://blast.ncbi.nlm.nih.gov/Blast.cgi. Nucleotide sequences obtained were compared with those available in the GenBank database which showed > 99% homology and > 95% coverage with *Candida albicans* in 7 cases (all except Patient no. 3) and *Citeromyces matritensis* in 1 case (Patient no. 3) [14]. Nucleotide sequences obtained from all 8 patients were submitted to NCBI with the respective accession numbers (Table 1).

**Table 1:** Results of laboratory tests, treatment given and outcome in all patients

Patient no.	KOH + CFW stain of corneal scraping	Gram stain of corneal scraping	Culture of corneal scraping	PCR of corneal scraping	ITS Sequence identification by BLAST (97–100%)	Genbank accession no.	Medical Treatment	Surgical treatment	Follow up period	Visual outcome at lst follow up
1	Round to oval shaped, structures + septate fungal fillaments	No organisms	No growth	Panfungal DNA positive	*Candida albicans*	OR911363	Amphotericin B	POST TPK GRAFT INFILTRATE	1month ( following up after in secondary center)	HM
2	Round to oval shaped, structures	Round to oval shaped, structures	*S. pneumoniae*	Panfungal DNA positive	*Candida albicans*	OR086088	Amphotericin B	TPK	5month	PL
3	Round to oval shaped, structures	Gram positive cocci	Coagulase negative staphylococcus	Panfungal DNA positive	Citeromyces matritensis	OR086087	Amphotericin B	None	1Month	20/25
4	Round to oval shaped, structures	No organisms	Not done	Panfungal DNA positive, panmicrosporidial negative	*Candida albicans*	OR752039	Gatifloxacin	None	4month	20/125
5	Round to oval shaped, structures	No organisms	No growth	Panfungal DNA positive	*Candida albicans*	OR911359	Amphotericin B	AC wash, intracameral amphotericin B, TPK	1month (lost to fu after tpk)	HM
6	Round to oval shaped, structures	Budding yeast	*S. epidermidis*	Panfungal DNA positive	*Candida albicans*	OR911364	Amphotericin B	None (post DMEK eye)	1month	20/100
7	Round to oval shaped, structures	No organisms	No growth	Panfungal DNA positive	*Candida albicans*	OR752040	Moxifloxacin	None	1 week	20/200
8	Round to oval shaped, structures	Round non-specific structures	*Corynebacterium* species	Panfungal DNA positive	*Candida albicans*	OR911362	Natamycin, Amphotericin B	None	6month	Lost to follow up

KOH + CFW-potassium hydroxide with calcofluor white, PCR = polymerase chain reaction, TPK-therapeutic penetrating keratoplasty, AC-Anterior chamber, HM- Hand movement, PL-Perception of light,

### Treatment

Medical treatment consisted of topical amphotericin B (Jolly pharma, 0.15%, prepared in Aurosol) as first-line drug. Natamycin %, gatifloxacin % and moxifloxacin% were some of the other drugs used. When required, surgical treatment consisted of therapeutic penetrating keratoplasty (TPK). Was other procedures (TABCL, Intracameral/intrastromal Ampho B) performed.

## Results

### Demographics

**Fig. 1:**
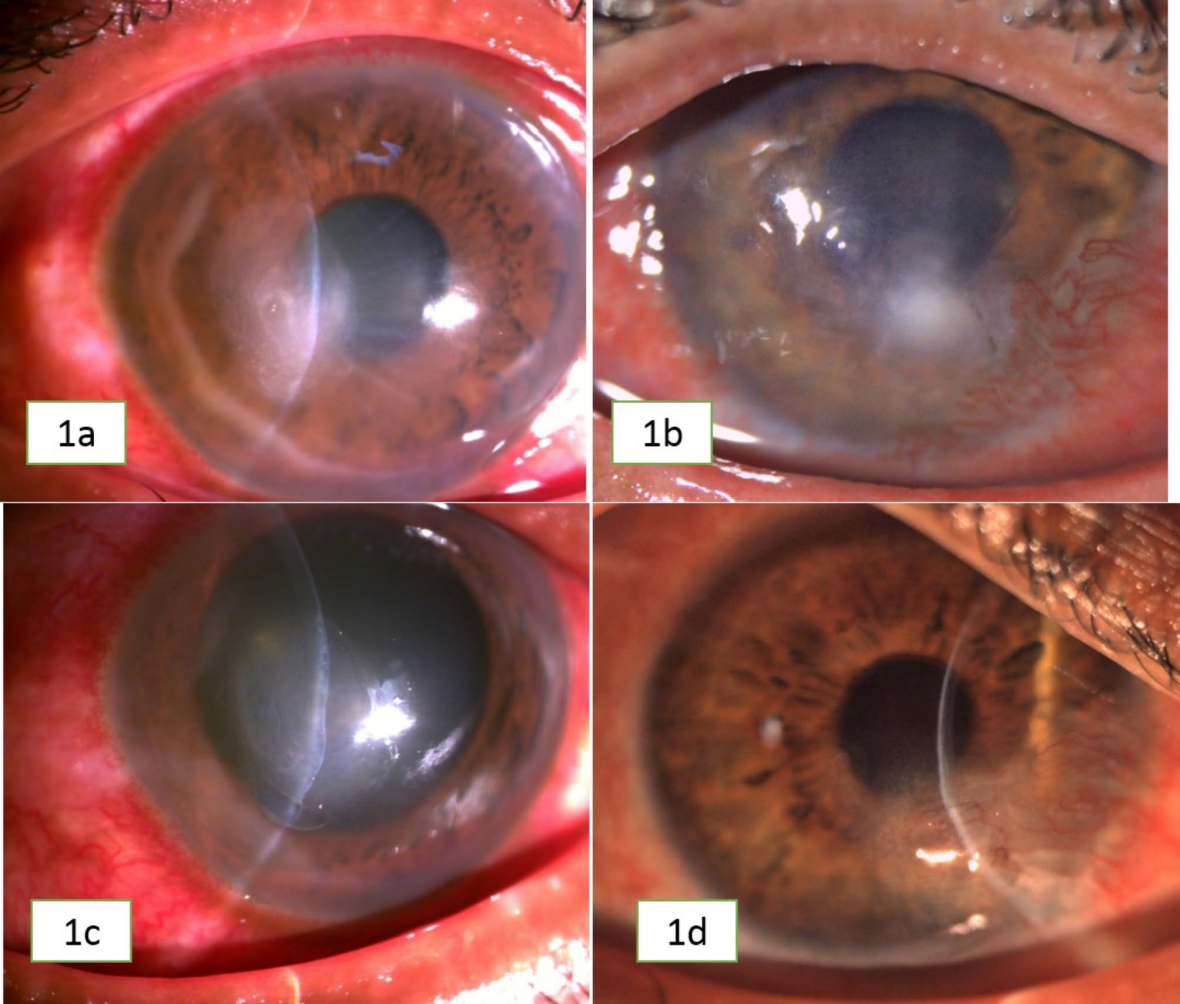
**1a** Clinical picture of patient-3 with superficial stromal infiltrate and surrounding ring infiltrate; Fig. **1c** Clinical picture of patient-3 after treatment which shows scaring; Fig. **1b** Clinical picture of patient-4 showing deep stromal infiltrate with surrounding vascularization; Fig. **1d**: Clinical picture of patient-4 after treatment which shows scaring

Clinical details of all cases is shown in Table 2. Five out of eight (63%) patients were male and rest three (37%) were female. The age of the patients ranged from 8 to 81 years (mean: 51.6 years +/- SD??). Right eye was involved in 5 (63%) patients. History of trauma with stone, mud, insect, battery; vegetative matter, was present in 5 (63%) patients (Patient no 1–5) and history of cataract surgery in one patient (13%) (patient no 6); history of corneal surgery in 2 patients (patient1,TPK and patient 6,DMEK), history of topical steroids use before presentation was reported in two patients (patient 6 and 7) and uncontrolled diabetes mellitus in one patient (patient 6). The duration of presentation ranged from 1 to 4 months with a mean of 17 days. The presenting visual acuity was worse than 20/200 in 4 (50%) patients. Four patients had grade 1, one patient had grade 2 and three patients had grade 3 ulcer at presentation. The ulcer was perilimbal in five patients, central in location in two and total corneal stromal infiltrate with melt in one patient. Two patients also had white hypopyon around 1–2 mm in size. **(**Fig. 1a and c represents pre-treatment and post treatment clinical picture of patient-3; **1b and 1d** represents pre-treatment and post treatment clinical picture of patient of patient-4 respectively.)

**Table 2:** Clinical details of eight cases that showed atypical yeast-like structures in the corneal scraping

Patient no.	Age/ Gender	Eye involved	Visual acuity at presentation	Past history	Duration of symptoms prior to presentation	Size of stromal infiltrate (mm)	Ulcer Grade	Depth of stromal infiltrate	Hypopyon (mm)	Clinical diagnosis
1	63 M	OD	PL	Trauma with stone, TPK 2 years back h/o Arthritis	1 month	3 × 2	1	Deep	2	Fungal (tpk done before for micrsporidial)
2	78 M	OD	PL	Fall of mud	10 days	Total corneal	3	Deep	NA	Microbial (FUNGAL)
3	41 M	OD	20/60	Insect fall	1 week	2.5 × 3, incomplete ring infiltrate	1	Mid stromal	NO	Acanthamoeba
4	42 M	OD	20/125	Trauma with battery	3 months	Central corneal cellularity with edema, partial LSCD	2	Mid stromal	no	Microsporidial
5	44 F	OS	PL	Trauma with vegetative matter	4 months	6 × 4	3	Full thickness (perforated)	< 1	Fungal
6	56 M	OS	20/100	Cataract surgery 2 months back, DMEK, history of steroid use, DM	5 days	2.5 × 2	1	Superficial	no	Microbial
7	8 F	OD	20/20	History of steroid use	1 week	3 × 2	1	superficial	no	Microbial
8	81 F	OS	PL	None	1 year	8 × 9	3	Deep stromal	no	Fungal

PL-Perception of light, M-male, F-female, OD-right eye, OS-left eye

Clinically, prior to microbiology evaluation, three patients were diagnosed as fungal keratitis, one each as *Acanthamoeba* keratitis and Microsporidial keratitis while three were considered non-specific microbial keratitis.

### Microscopy, culture, PCR and DNA sequencing assays

**Fig. 2:**
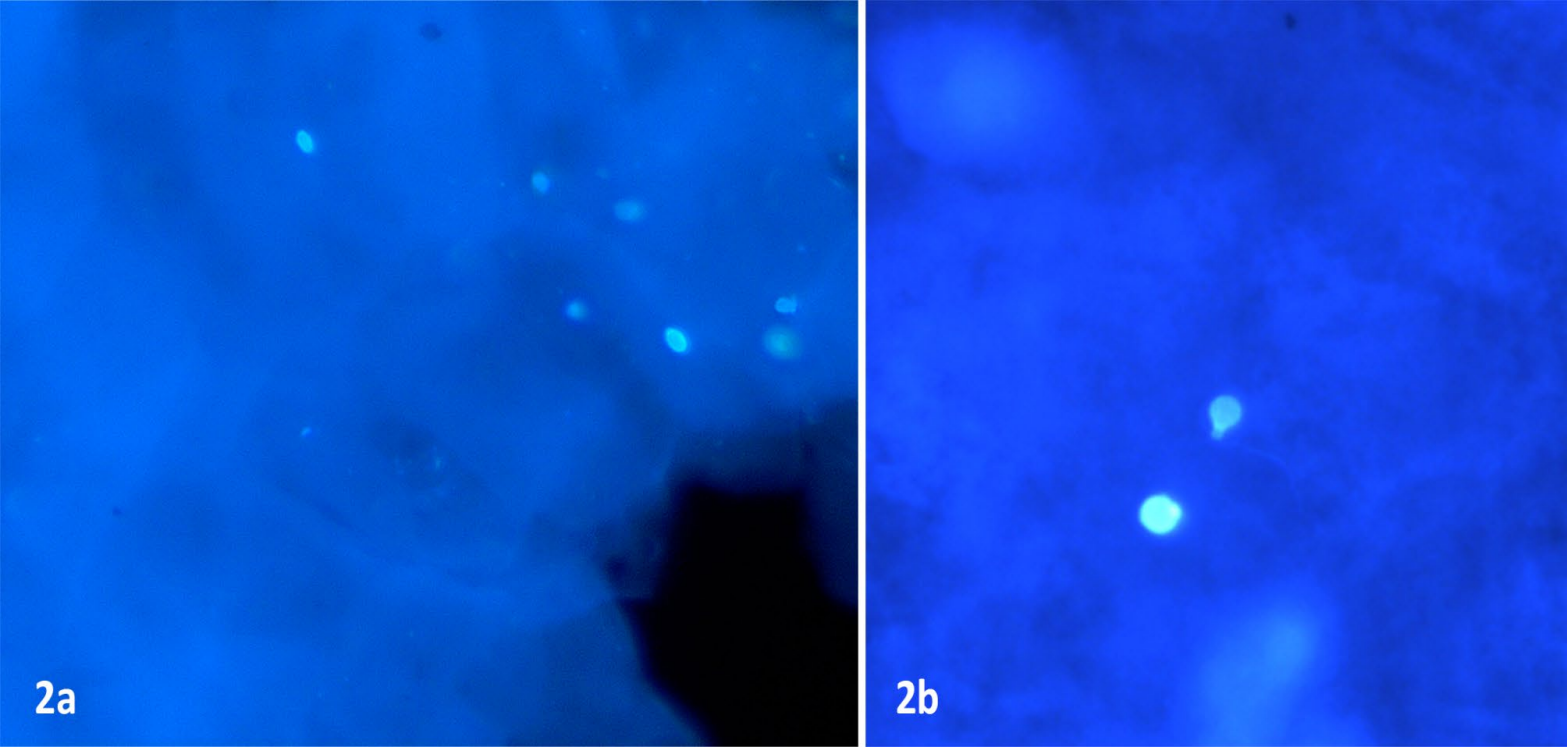
Representative pictures demonstrate microscopy results of corneal scraping of patients with KOH + CFW mount (**A**) Patient number 3: oval brightly fluorescent cells with no budding (**B**) Patient number 5: Round fluorescent spore with slightly protruding structure

Figure 2a and b show (patient number 3) the fluorescent atypical structures that were round to oval shaped, without any budding or pseudo-hyphae or any germ tube but with a hyperfluorescent point at one of the poles (x400 total magnification, KOH + CFW mount). These structures were not visible in corresponding Gram stained smears of the corneal scraping from these patients except for patient number 2 and 6. Patient number 8 showed non-specific round bodies in the Gram stain of the corneal scraping. These structures gave an impression of either artifacts or microsporidial spores or yeast-like fungal spores as per our past experience in ocular microbiology (SS-more than 30 years, HSB- 5 years). Corneal scrapings from none of the cases showed yeast growth even after 14 days of incubation, although insignificant growth of bacteria was seen in 4 cases (Table 1).

**Fig. 3:**
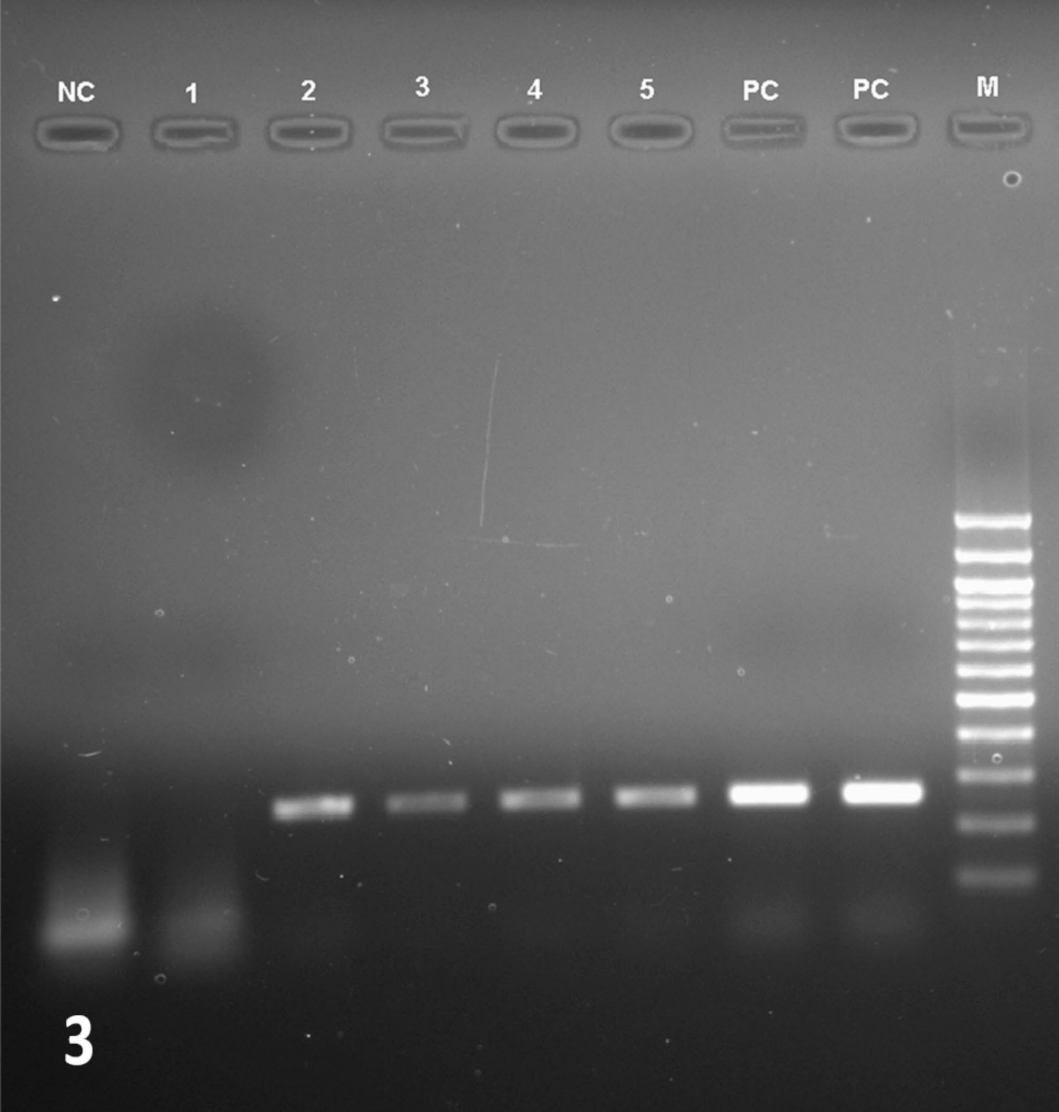
Agarose gel electrophoresis picture demonstrates ITS based panfungal PCR amplified products from 5 patients (256 bp) along with positive and negative control and 100 bp DNA ladder. Label the figure with bp size

**Fig. 4:**
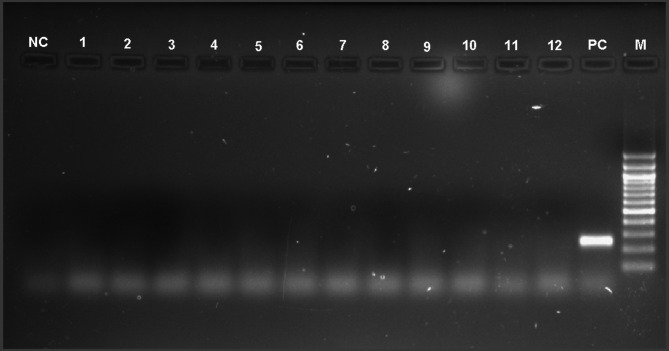
Agarose gel electrophoresis picture demonstrates no amplification in ITS based panfungal PCR from corneal scrapings of 12 control patients. Positive and negative control along with 100 bp DNA ladder are also seen. Label the figure with bp size

Fungal DNA was amplified from the DNA of corneal scraping of all eight cases in conventional PCR **(**Fig. 3**)**. Sanger sequencing followed with BLAST analysis of all amplicons identified *Candida albicans* in seven and *Citeromyces matriensis* in one. Nucleotide sequences were submitted to NCBI with accession numbers **(**Table 1**).** No amplification was seen from the amplified products of control samples while positive and negative controls were satisfactory (Fig. 4).

### Response to treatment

All patients were treated with antifungal drugs, topical 0.15% amphotericin B/ 5% natamycin. Patient number 5 required anterior chamber wash and intracameral amphotericin B as the infection spread to the anterior chamber. Patient number 2 required urgent TPK as it was a large infiltrate extending to limbus. Two patients could not be given antifungal treatment as case 4 did not take the prescribed drug and in case 7 the diagnosis was made in second visit. However, the ulcer was found healed on follow up in both cases. Treatment outcome for all patients is summarised in Table 1. While three patients required therapeutic penetrating keratoplasy, others healed with scarring. The average period of follow up was 1.6 months. While best corrected visual acuity at the last follow up was better than 20/200 in 4 patients, 3 patients ended up with poor visual acuity and 1 patient was lost to follow up.

## Discussion

Microscopic evaluation of the corneal scrapings is one of the most rewarding and rapid methods for the etiological diagnosis of microbial keratitis aiding in early initiation of specific treatment. A large body of literature supports the application of this methodology in cornea clinics engaged in treatment of microbial keratitis patients [15, 16]. However, the limitations of the method include low sensitivity and requirement for high expertise [12]. Experience of the observer is one of the key players that determines accuracy of the results. The ocular microbiologists involved in the microscopic observation of the corneal scrapings in these cases even with their experience were uncertain about the fluorescent structures in KOH + CFW mount that resembled yeast or microsporidial spores. Some of the cells had a hyper fluorescent spot at one of the poles. An earlier study reported that, Golgi complex cluster towards one end of cell prior to cell division that might be visible as brighter fluorescent spot in KOH + CFW mount [10]. The budding seen in some of the cells were broad based and not typical of budding yeast. Usually budding yeast cells have clear budding shape similar to figure “8”, with a large mother cell and small daughter cell. Daughter cell can vary in its size based on origin, i.e., daughter cell derived from an old mother cell is comparatively larger, than the one derived from a young mother cell [17].

Normally, the KOH + CFW mount of corneal scraping is examined under 40X objective lens. Calcofluor white is a fluorescent brightener that has a high affinity to bind cellulose present in the cell wall of fungi, yeast and microsporidia. Use of CFW along with KOH increases the diagnostic positive rate, sensitivity and specificity of smear examination and simultaneously reduces the diagnostic time. A small fragment of any fungal hyphae or few yeast / Microsporidia cells can be noticed in KOH + CFW mount, that may be missed with only KOH mount. Hence, KOH + CFW has a potential value for clinical applications. In our recent experience, we saw a much clearer view of these atypical structures at higher magnification (100X objective lens in KOH + CFW mount). Therefore, we suggest the use of 100x objective lens for better visualisation of such atypical structures.

Absence of these atypical structures in corresponding Gram stain may be attributed to relatively lesser sensitivity of Gram stain compared to KOH + CFW for the detection of fungal spores and other pathogens such as *Microsporidia* or *Acanthamoeba* in clinical samples [18, 19]. It is also possible that unlike Gram stain, use of KOH causes clearing of the background tissue making these structures stand out prominently. Inability to grow in culture could be due to either low numbers of the organisms or non-viable organisms.

In recent times, molecular methods (conventional PCR, real time PCR, DNA sequencing, next generation sequencing etc.) have found their way into clinical diagnostic laboratories for detection and identification of microorganisms in clinical samples [20, 21]. They are extremely versatile and apart from accurate and specific identification, they help to recognise previously unknown organisms [22]. Strong clinical suspicion of yeast prompted us to proceed further with PCR followed by sequencing to confirm the identity of these structures as *Candida* spp. and *Citeromyces matriensis*.

Our results emphasise that KOH + CFW stain can demonstrate atypical spore like structures (with or without budding) in corneal scrapings that can be suggestive of *Candida* species. In absence of growth in culture they can be further confirmed by ITS targeted pan fungal PCR. The clinical importance of this understanding was shown by the fact that, out of 6 cases given antifungal therapy all cases responded to targeted treatment except one which had already extended to limbus requiring TPK.

Absence of detectable *Candida* DNA in all 12 corneal scraping samples, from smear and culture negative cases, suggests that *Candida* is unlikely to be a normal flora in the cornea and could not have been a contaminant in the samples of the eight cases reported in this series. This is in contrast to a recent report that has shown *Candida* DNA in normal conjunctiva by next generation sequencing (NGS) [23]. However, the difference in the sensitivity of the PCR technology in the two studies would explain the discrepancy. Using NGS for diagnosis in a clinical scenario remains far-fetched at this point in time.

Clinically, *Candida* keratitis may resemble bacterial keratitis [24]. Regina et al. in their study on characteristics of *Candida* keratitis reported that, out of 29 patients, keratomycosis was suspected in 2 patients and rest were considered bacterial keratitis [25]. Therefore, to avoid erring in favour of bacterial keratitis, we recommend increased awareness among microbiologists to report this entity for the early initiation of antifungal treatment.

In this series topical amphotericin B 0.15% was used in all except two patients for treatment. In general, its use is limited because of unavailability of ready to use topical preparation and because it needs compounding pharmacy for formulation from injectable form. It is also associated with surface toxicity leading to punctate corneal erosions, stromal edema and iritis. Therefore, topical natamycin and voriconazole are other alternatives for treating *Candida* keratitis [25].

The limitation of the study was its retrospective design, small sample size and involvement of several treating clinicians that may account for the final visual and functional outcomes in patients according to their clinical decisions.

We conclude that the awareness of considering round to oval shaped atypical structures, without any budding or pseudo-hyphae with a hyperfluorescent point at one of the poles in KOH + CFW mount of corneal scraping of a suspected microbial keratitis patient as budding yeast, will be helpful in clinical practice. Further confirmation can be made by fungal PCR.

## Data Availability

No datasets were generated or analysed during the current study.
